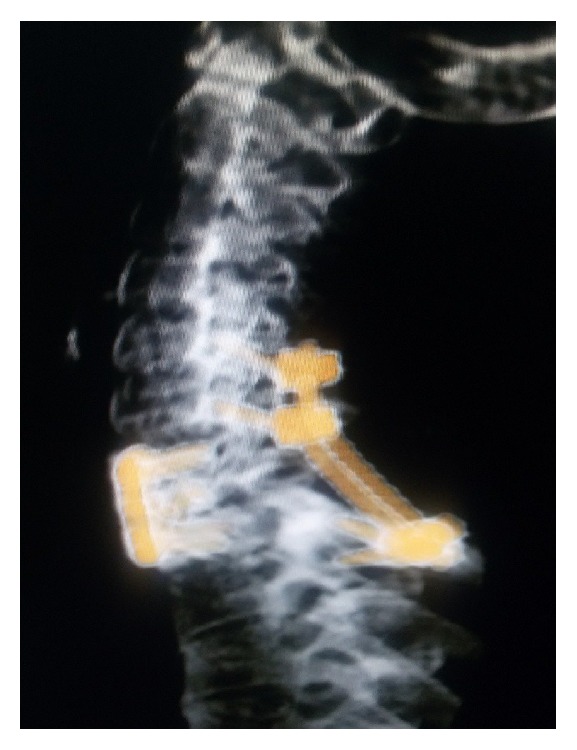# Traumatic Cervical Spondyloptosis in a Neurologically Stable Patient: A Therapeutic Challenge

**DOI:** 10.1155/2015/540919

**Published:** 2015-07-16

**Authors:** Sunil Munakomi, Binod Bhattarai, Iype Cherian

**Affiliations:** International Society for Medical Education, College of Medical Sciences, P.O. Box 23, Chitwan, Nepal

## Abstract

This is a case report of a neurologically intact patient following posttraumatic cervical spondyloptosis. We discuss the disease, management protocol and some surgical nuances to prevent any damage to the cord during different stages of its treatment.

## 1. Introduction 

Intact neurological status in a case of cervical spondyloptosis is a rare entity with only few cases being reported so far [1–3]. The main dilemma that remains is which approach can be taken for the management of cervical spondyloptosis. Initially with traction, intraoperatively while positioning and later on during reduction and stabilization procedures, damage to the cord, the nerve roots, and vertebral artery can inadvertently lead to neurological deficits in such patients. Therefore the upmost care needs to be taken during management especially when the patient is neurologically intact [2]. Herein we discuss the management of one such rare entity.

## 2. Case Report

**Figure 1:**
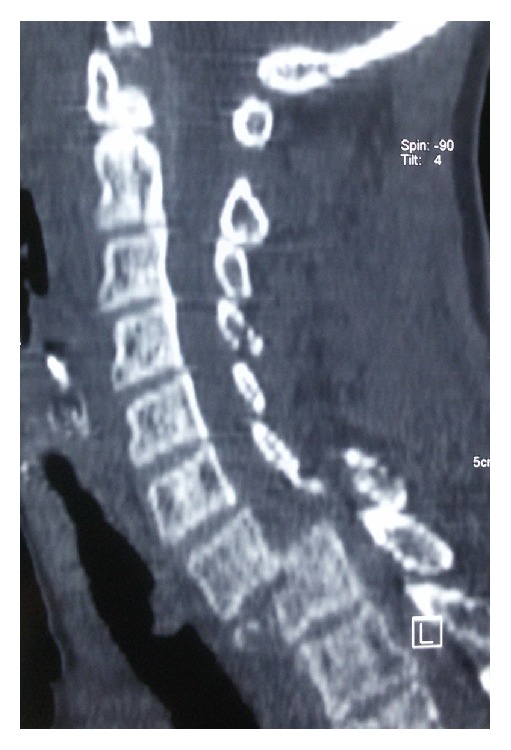


**Figure 2:**
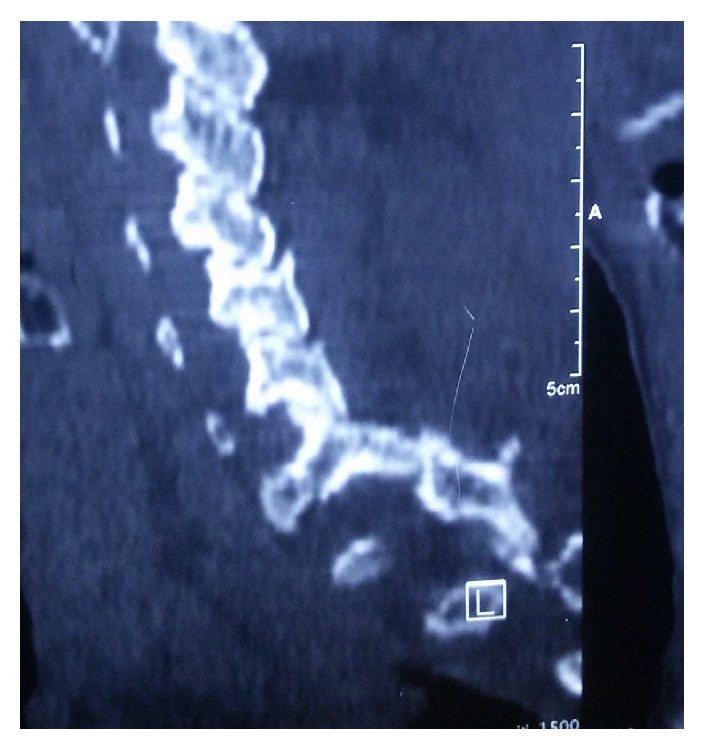


A fifty-six-year-old female resident from a remote village in Nepal presented to our emergency six hours after a fall from a tree which she had climbed to collect food for her livestock. She was taken to a district hospital. The X-ray of cervical spine showed cervical spondyloptosis and she was referred to our tertiary care centre. At the time of presentation in the ER room, her Glasgow coma scale was 15/15 and she was neurologically intact. General systemic examination was normal. She was complaining of neck pain so a rigid cervical collar was placed. Her single breath count (SBC) was >20 and there was no evidence of Horner's syndrome. As the previous X-ray showed some doubt in the lower region of her subaxial spine, we investigated with the CT scan her cervical spine which confirmed cervical spondyloptosis at C7-T1 region (Figure 1). She was started on methylprednisolone as per the national acute spinal cord injury study (NASCIS III) protocol. Gradual traction with continued neurological examination was applied to her and X-ray was checked to prevent overdistraction. Despite the application of recommended weight there was no evidence of reduction which probably was because of bilateral locked facet joints (Figure 2).

**Figure 3:**
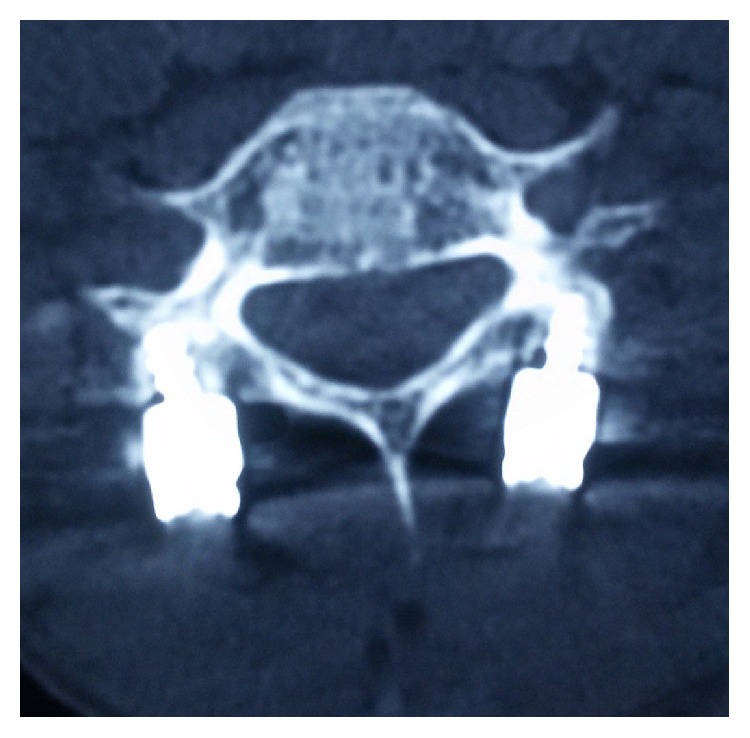


**Figure 4:**
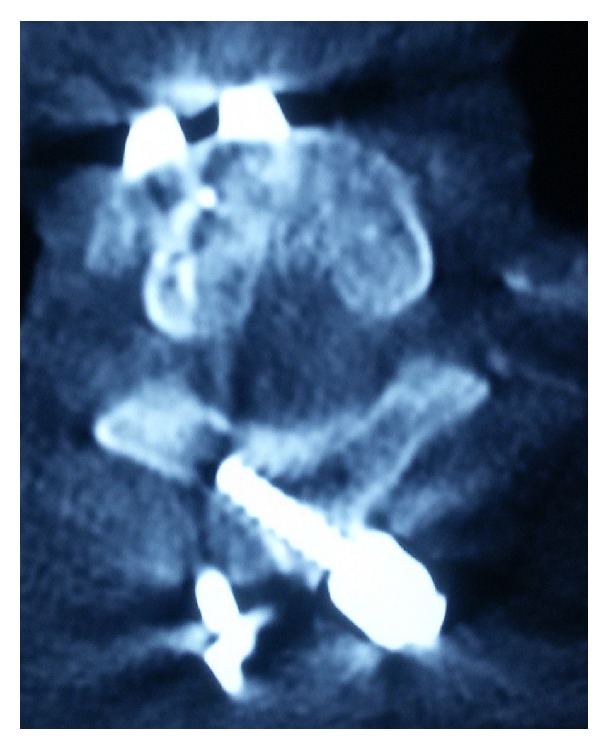


**Figure 5:**
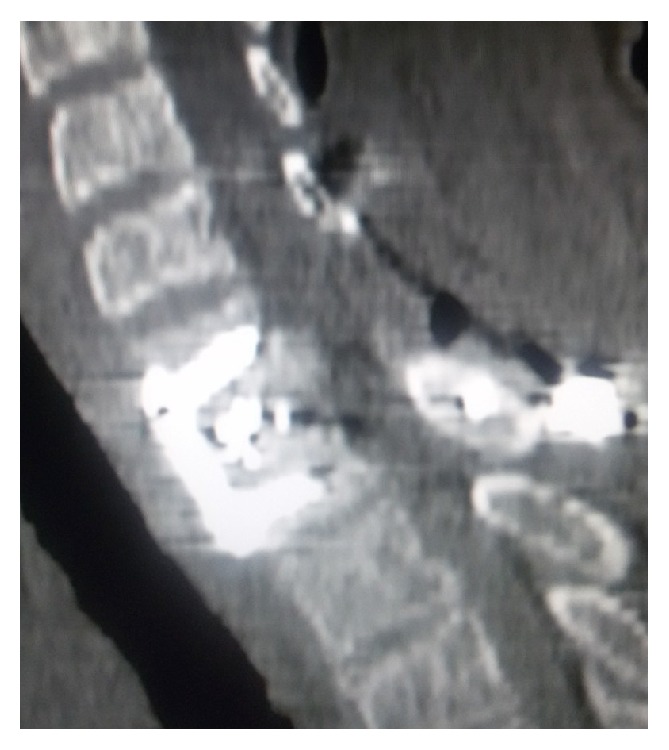


The decision was taken for manual reduction under anesthesia followed by global fixation with a view of providing rigid stable construct to a mobile cervical segment (cervicothoracic junction). An anterior cervical discectomy was done; then the posterior approach was taken. Fractures of the spinous process and lamina of C7 were confirmed. Reduction of the locked facets with alignment of the anterior and posterior spinal lines was confirmed by C-arm. Lateral mass screws were placed at C6; crossed laminar screws were placed at T1 and were interconnected with rods (Figures 3 and 4). Then the patient was placed supine with placement of cage at C7-T1 followed by C7-T1 plating and screw fixation (Figure 5).

**Figure 6:**
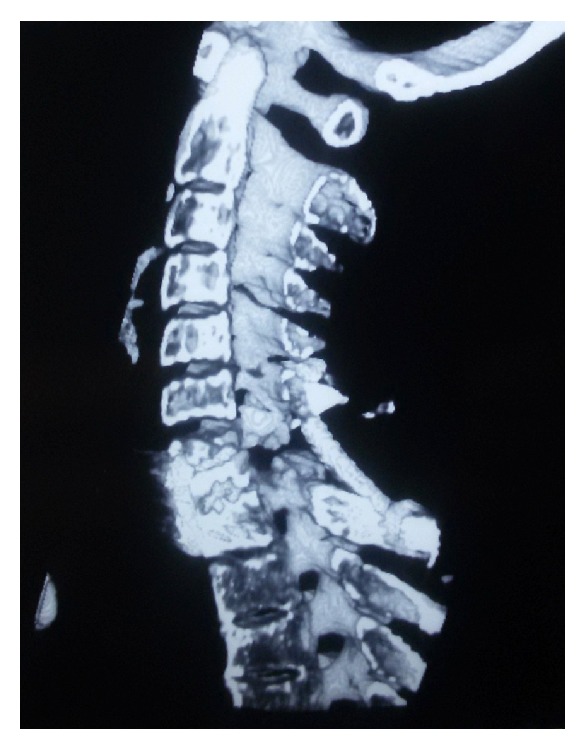


Postoperatively patient had no deficits and no Horner's syndrome. Checked CT of cervical spine showed good reduction of the spondyloptotic segment (Figures 6 and 7). Patient was advised bed rest for 4 weeks and then gradually mobilized on cervical collar. Patient made an uneventful full recovery.

## 3. Discussion

Spondyloptosis is a condition where there is a complete slippage of one vertebral body over the corpus of the adjacent one [3]. It is more common on the lumbar segment with only a few cases of subaxial spine spondyloptotic cases being reported so far in the literature [2, 3].

Patient invariably has some features of myelopathy or quadriparesis because of the compression of the cord. Sometimes when there is a fracture of the posterior elements, then, due to spontaneous decompression, some of the patients may escape such disastrous consequences [1].

If the patient has poor neurological status (Frankel A) then the aim of the management is to provide reduction and support which we can achieve from anterior approach. The dilemma appears in the management of those few who are functionally good. In them we opt for traction taking care not to overdistract the segment [4]. Since it is in a mobile segment there is a need of a solid construct after proper reduction for which global fixation is justified. Literature justifies taking anterior approach for discectomy so as to prevent damage from the prolapsed disc during the prone position of the patient [5]. Then the posterior approach is taken with reduction of the locked facets, confirmation of the realignment via C-arm followed by lateral mass or cross laminar screw and rod fixation. Posttraumatic dural tear and damage to the vertebral artery should be borne in mind. Some author advocates posterior approach so as to prevent the risk of graft dislodgement if anterior approach is chosen initially [3].

## 4. Conclusion

Intact neurological status despite posttraumatic subaxial cervical spondyloptosis is a rare entity. Upmost care needs to be governed and correct surgical algorithm needs to be sought for the management of such cases.

## Figures and Tables

**Figure 7:**